# Modeling the Structure of Crystalline Alamethicin and Its NMR Chemical Shift Tensors

**DOI:** 10.3390/antibiotics10101265

**Published:** 2021-10-18

**Authors:** Jiří Czernek, Jiří Brus

**Affiliations:** Institute of Macromolecular Chemistry, Czech Academy of Sciences, 16206 Prague, Czech Republic; brus@imc.cas.cz

**Keywords:** antimicrobial peptides, alamethicin, solid-state NMR, DFT

## Abstract

Alamethicin (ALM) is an antimicrobial peptide that is frequently employed in studies of the mechanism of action of pore-forming molecules. Advanced techniques of solid-state NMR spectroscopy (SSNMR) are important in these studies, as they are capable of describing the alignment of helical peptides, such as ALM, in lipid bilayers. Here, it is demonstrated how an analysis of the SSNMR measurements can benefit from fully periodic calculations, which employ the plane-wave density-functional theory (PW DFT) of the solid-phase geometry and related spectral parameters of ALM. The PW DFT calculations are used to obtain the structure of desolvated crystalline ALM and predict the NMR chemical shift tensors (CSTs) of its nuclei. A variation in the CSTs of the amidic nitrogens and carbonyl carbons along the ALM backbone is evaluated and included in simulations of the orientation-dependent anisotropic ^15^N and ^13^C chemical shift components. In this way, the influence of the site-specific structural effects on the experimentally determined orientation of ALM is shown in models of cell membranes.

## 1. Introduction

The current status of the antimicrobial resistance problem was summarized in an ominous *Nature* editorial from July of this year [1]. This problem has been studied for decades [2], and one of the ways of tackling it is through the development of peptide-based antibiotics (see the most recent reviews [3,4] and references cited therein). Relevant antimicrobial peptides (AMPs) interact with cell membranes in various disruptive ways, which were most recently outlined in reference [5]. A number of experimental techniques was used in attempts to understand the details of those interactions [6,7] and, at present, four main models of membrane-damaging AMP activity are considered [4] (see also reference [8]). It should also be mentioned that molecular dynamics simulations were extensively applied to model interactions between AMPs and lipid bilayers, as summarized in reference [9]. Moreover, the applications of a variety of computational techniques to AMPs were recently reviewed [10].

Peptaibols of the alamethicin (ALM) family [11] are among the most frequently studied AMPs [12]. ALM is a key molecule when investigating pore formation by peptides in interfacial membranes [13]. Important results regarding the behavior of ALM in membrane mimetic environments were obtained by the solid-state NMR (SSNMR) measurements (see references [14,15] for recent reviews of these experimental approaches). The Bechinger group analyzed the orientation-dependent ^15^N and ^31^P SSNMR spectra of ALM F50/7 (see Section 2.1 for a structural description of ALM peptaibols) that was uniformly labeled with ^15^N isotope [16]. On the basis of the ^2^H and ^15^N SSNMR experiments, carried out for an Aib8 (α-aminoisobutyric acid in the position 8 of ALM backbone) residue labeled with either ^2^H or ^15^N, the Vosegaard group provided dynamic information about this site in ALM F30 [17]. The Naito group prepared as many as eleven derivatives of ALM with the methyl ester group at the *C*-end, which were singly labeled with ^13^C at the respective carbonyl carbon position, to describe the carbonyl ^13^C chemical shift oscillations [18] and interpret them in terms of peptide topology in lipid bilayers [19]. It should be noted that the aforementioned SSNMR investigations were later put into a wider context in references [20,21,22]. At the time these experiments were performed (about 12 years ago in the case of studies [16,17]), it was practically impossible to supplement them with the results of the quantum chemical calculations of the structural and spectral parameters of relevant ALM models. Since then, huge progress has been made in two connected directions. The first area is called NMR crystallography, and was most recently reviewed by Hodgkinson [23]. X-ray diffraction (XRD) and SSNMR experimental studies can be usefully complemented by plane-wave density-functional theory (PW DFT)-based calculations of periodic solids to accurately characterize structures and SSNMR spectral parameters [24]. The second area concerns well-known improvements in related software and hardware, which make it possible to routinely compute the properties of structures with a crystal unit-cell volume exceeding five nm^3^ (if the number of symmetry-independent molecules in such a unit cell is not excessively large) [25]. The aforementioned development enabled us to model crystalline ALM. 

Our modeling was focused on the desolvated form of ALM, whose structure was approximated based on coordinates of one of the crystallographically independent molecules of ALM F30 from the XRD study [26], as described in Section 2.1. There are two main results of this work. The first one is the fully optimized solid-phase structure of ALM, which was used to reliably predict values of the SSNMR parameters of the key {^15^N, ^13^C} nuclei of peptide groups to provide an additional insight into some of the experimentally observed trends. Based on this, the second main result of our study was obtained: conclusions about the variability of these SSNMR data along the ALM backbone were drawn, and the influence of this variability on the measured parameters was described. Simulations of the relevant anisotropic chemical shift components as a function of orientational parameters were performed for the ^15^N amidic and ^13^C carbonyl sites of ALM. We thus presume that the PW DFT methodology will soon be applied to other AMPs during SSNMR studies of their mechanism of action in the discovery process of new antibiotics.

## 2. Results and Discussion

### 2.1. The ALM Structure 

**Figure 1:**
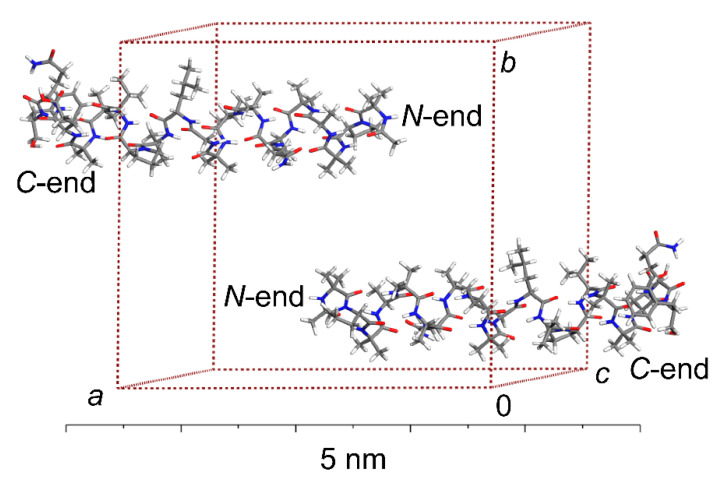
The crystallographic unit cell of ALM-18 model. Color-coding of the sticks: white, grey, blue, and red for hydrogen, carbon, nitrogen, and oxygen atoms, respectively.

Alamethicins contain 20 amino acid residues and belong to subfamily 1 of peptaibols [27]. Their isolation from fungus and the subsequent characterization of resulting mixtures was studied in detail [11]. Most recently, the three major amino acid sequences of ALM were denoted as E18, A6 and U6 [28], which is a more convenient classification than the one based on the retention factor value possibly followed by fraction designation. Hence, the ALM structure considered in the present work is termed ALM-E18. It is emphasized that in E18 primary structure of an ALM peptide, there is Ala present in the position 6 (would be Aib in U6), and Glu in the position 18 (would be Gln in A6). ALM-E18 thus comprises the following fragments: 

Ac-Aib1-Pro2-Aib3-Ala4-Aib5-Ala6-Gln7-Aib8-Val9-Aib10-Gly11-Leu12-Aib13-Pro14-Val15-Aib16-Aib17-Glu18-Gln19-Phl20

where Aib and Phl, respectively, refer to α-aminoisobutyric acid and phenylalaninol, and all chiral amino acids are L stereoisomers. The periodic model of ALM-E18 was created using coordinates of the “chain C” molecule from the XRD study [26] (the Protein Data Bank entry: 1AMT) and fully optimized by the PW DFT approach, which is detailed in Section 3. It should be noted that the asymmetric unit of the XRD structure [26] also contained two acetonitrile and 13 methanol molecules, but they are not considered here. The CIF file with the PW DFT structure is included in the Supporting Materials. In brief, the crystal unit cell belongs to the monoclinic *P*2_1_ space group and has a large volume of over 20 nm^3^ while containing two symmetry-related ALM molecules packed in an antiparallel arrangement (see Figure 1). This structure is representative of a solvent-removed crystalline ALM, which could be active through the carpet mechanism. In this mode of action, no interactions between AMPs are required, and AMP molecules should extensively cover a membrane to form micelles (for a recent discussion, see references [6,29,30]).

**Figure 2:**
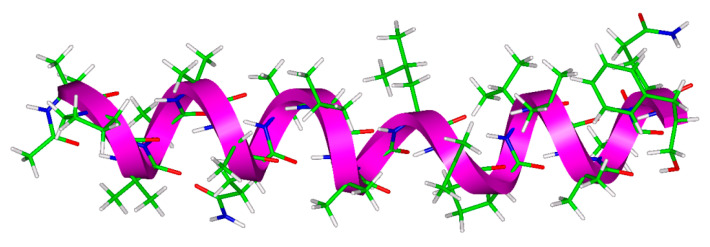
The asymmetric unit of ALM-18 model, shown together with an irregular helix. Color-coding of the sticks: white, green, blue, and red for hydrogen, carbon, nitrogen, and oxygen atoms, respectively. The helix is depicted as magenta solid ribbon.

It is well-known that the dry ALM assumes a right-handed helical conformation. Figure 2 shows the irregular helix of an ALM-E18 molecule, clipped out of the crystal structure after its PW DFT optimization. Table 1 summarizes the key structural features, namely, the values of Ramachandran angles and the hydrogen-bonding involvement of respective sites, while any details can be found in the PDB file included in the Supporting Materials. It is worth mentioning that a short segment of the 3_10_ helix formed by residues toward the *C*-end was described in a number of studies, which investigated ALM in membrane environments and in organic solutions [31]. In the present ALM-E18 model, however, its tertiary structure is predominantly the α-helix, which is broken by Gly11. An inspection of Table 1 shows that a majority of hydrogen bonds are of the i→i+4 type, and most [φ;ψ] values and  |φ+ψ| sums are typical for an α-helix. The presence of the 3_10_ helix might be inferred from the following hydrogen bonds, of i→i+3 type: Val9 → Leu12, Aib10 → Aib13, and Leu12 → Val15, but this would not be supported by values of the corresponding [φ;ψ] dihedral angles (see Table 1). Certain parameters of the ALM helix are discussed in the next section. 

### 2.2. The ^15^N SSNMR Parameters of Amidic Nitrogens

The ^15^N NMR chemical shift tensor (CST) of amidic nitrogens is the key physical quantity used in studies of the spatial orientation of an investigated helical peptide in planar membranes. The orientation for such a peptide can be expressed in terms of angles ρ, of the azimuthal rotation of each amino acid site, and τ, of the tilt of each helical fragment; τ is taken with respect to $\vec{n}$, that is, the direction perpendicular to the plane of a membrane [32]. In a typical experimental approach, the sample is oriented so that $\vec{n}$ and the direction of an external magnetic field coincide, and the $\delta_{∥}$ component of the ^15^N CST is measured and analyzed to arrive at τ value. This analysis is straightforward, because $\delta_{∥}$ is assumed to be approximately parallel to the helix axis ($\delta_{∥}\;$ is the rotationally averaged direction of $\delta_{33}$, which is one of the principal elements of the CST: $\delta_{11},\;\delta_{22},\;\delta_{33}$ with $\delta_{11}\leq \delta_{22}\leq \delta_{33}$, while the isotropic component, $\delta^{\mathrm{iso}}$, of the CST is $\delta^{\mathrm{iso}}=\left(\delta_{11}+\delta_{22}+\delta_{33}\right)/3$). Importantly, the values of τ were previously obtained for ALM-A6 in reference [33] for a number of structural models and by employing several sets of the CST data. The PW DFT calculations can supplement such analyses, as they reveal a variation in the ^15^N CSTs along the ALM backbone. It is, therefore, possible to evaluate the effect of that variation upon the extracted tilt angle. First, however, the ^15^N CST principal elements have to be obtained using their theoretical counterparts, which are the principal elements of the ^15^N chemical shielding tensor, $\sigma_{11},\;\sigma_{22},\;\sigma_{33},$ with $\sigma_{11}\geq$ $\sigma_{22}\geq \;\sigma_{33}$. Here the conversion is achieved using the theoretical chemical shift, ε, defined in our previous work [34,35] (in analogy with δ  data, $\varepsilon_{11}\leq \varepsilon_{22}\leq \varepsilon_{33}$, and of course $\varepsilon^{\mathrm{iso}}=\left(\varepsilon_{11}+\varepsilon_{22}+\varepsilon_{33}\right)/3$). The parametrization is based on completely reliable structures and single-crystal SSNMR measurements of the CSTs for a set of small peptides [24] and, in shorthand notation, is: $\left\{\varepsilon_{pp}\right\}=-0.93574\times \left\{\sigma_{qq}\right\}+209.54\;$ ppm, where $\left\{\varepsilon_{pp}\right\}$ and $\left\{\sigma_{qq}\right\}$ denote corresponding values of the theoretical chemical shift and the chemical shielding components, respectively. Thus, the calculated ε data that are shown in Table 2 would approximate the site-specific δ data if these were available from the experiment. The accuracy of this approximation appears to be of several ppm for each of the CST components on the basis of a comparison for Aib8. Namely, the Vosegaard group obtained $\delta_{pp}$ values of {66.7, 81.3, 230} ppm for this site (reported using the Haeberlen notation [36] in reference [17]), while the corresponding $\varepsilon_{pp}$ values are {68.5, 78.6, 236.4} (see Table 2). The isotropic chemical shifts $\delta^{\mathrm{iso}}$ and $\varepsilon^{\mathrm{iso}}$ then become 126.0 and 127.8 ppm, respectively. 

**Figure 3:**
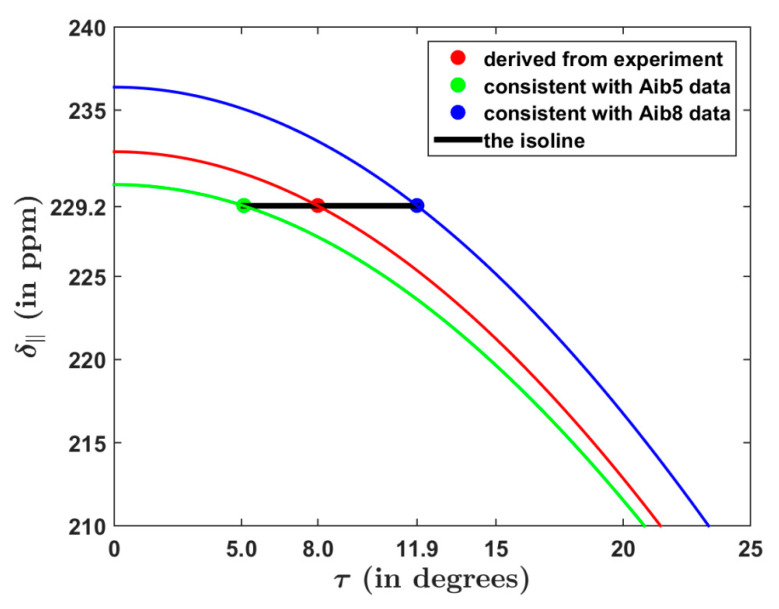
The dependence of the unique component of the ^15^N chemical shift tensor upon the tilt angle in the ALM-18 model described in the text. The expression for simulated lines is given in Section 3.2, while the underlying data are specified in Section 2.2.

The ^15^N SSNMR parameters introduced in the preceding paragraph were employed to directly assess how the determined τ angle would be influenced by a variation in the CST along the ALM backbone (see Figure 3). For one of the structural models, and for {64.5, 85.5, 232.5} ppm $\delta_{pp}$ values collectively describing Aib sites, the tilt angle of 8.0° was obtained for ALM-A6 by the Bechinger group [33]. Using Equation (1), shown in the Materials and Methods section, these values give an $\delta_{∥}$ of 292.2 ppm. When this $\delta_{∥}$ value is used together with the $\varepsilon_{pp}$ data of Aib5 in ALM-E18 model, the resulting τ becomes 5.0°, while the same $\delta_{∥}\;$ value leads to  τ of 11.9° if the $\varepsilon_{pp}$ data of the Aib8 residue are considered instead. Importantly, an angle between the helix axis (see Appendix A) and the direction of the $\delta_{33}$ of amidic nitrogens of Aib5 and Aib8 in ALM-E18 amounts to only 6.4° and 5.3°, respectively, as is required for this analysis to be valid. It should be noted that Aib5 and Aib8 are located in a regular α-helical segment of the ALM molecule (despite the differences between ALM-A6 and ALM-E18 structures). Even in this situation, however, the site-specific variation in the ^15^N CST is expected to lead to an uncertainty of several degrees in the extracted τ value. 

Further details of the predicted ^15^N CSTs can be found in Table 2. For the chemical shift data, the results agree with the known trend of the isotropic chemical shift and the principal element values, with all being systematically higher for Aib sites compared to the classical amino acids [33]. In the present case, the average values of $\varepsilon^{\mathrm{iso}}$, $\varepsilon_{11}$, $\varepsilon_{22}$, and $\varepsilon_{33}$ for Aib residues amount to 127.3, 68.3, 81.7, and 231.9 ppm, respectively. These averages are 115.1, 52.4, 73.8, and 219.2 ppm, respectively, for the proteinogenic amino acids other than the two prolines. However, this trend was previously confirmed by means of the PW DFT calculations in our study of Ampullosporin A [37]. The angles α, β, γ  specify an orientation of the ^15^N CSTs tensors in the crystal frame, and are defined in the Materials and Methods section. In short, angle α quantifies the departure of the $\delta_{33}$ component from the peptide plane, and is expected to be negligible [38]. Indeed, its values are all lower than 6° (see Table 2). The angle β is taken between the amide N–H bond vector and the $\delta_{33}$ component. This angle is crucial for the interpretation of SSNMR experiments correlating the amide N–H dipolar interaction with isotropic chemical shifts or chemical shift anisotropies [39]. The β values are higher for proteinogenic amino acids (19.2° ± 2.2°) than for Aib (13.7° ± 1.3°), as could be expected from previous PW DFT calculations [24]. The angle γ. describes how the ^15^N CST is slanted relative to the peptide plane. The γ values are completely site-specific [37], but they are given by the orientation of the $\delta_{22}$ component with respect to the peptide plane, and thus would undergo the rotational averaging with the $\delta_{11}$ component of a peptide in a membrane environment. 

### 2.3. The ^13^C SSNMR Parameters and the Chemical Shift Oscillations of Carbonyl Carbons 

As mentioned in the preceding section, a careful conversion of the computed principal elements of chemical shielding tensors to the corresponding theoretical chemical shift data is needed to obtain reliable estimates of the principal elements of the CSTs. Here, the experimental results for the ^13^C CSTs of carbonyl carbons were taken from scrupulous single-crystal SSNMR measurements by Takeda et al. [40] for three small molecules with accurately determined structures, which are specified in the Materials and Methods section, together with details of the PW DFT calculations. Both datasets are shown in Table S1 and Figure S1, and their correlation is described by $\left\{\varepsilon_{pp}\right\}=-0.99314\times \left\{\sigma_{qq}\right\}+172.50\;$ ppm with a standard deviation of 1.8 ppm and the adjusted *R*^2^ of 0.99918. This calibration is employed here to obtain the chemical shifts.

Table 3 summarizes the key ^13^C SSNMR data of the carbonyl carbons of ALM backbone. The isotropic chemical shift values fall into a typical range for α-helical fragments of polypeptides [41], with just one exception. That exception is the value predicted for Gly11, namely, 171.7 ppm. This value is not surprising, as it was found at the site where an irregular α-helix of ALM-E18 model was broken; hence, the carbonyl group is not involved in hydrogen bonding. The isotropic chemical shifts in the remaining residues lie in a narrow interval of 4.5 ppm (see Table 3). As anticipated, the principal elements of the predicted CSTs generally agree with the values for the α-helixes of peptides. For instance, Ala6 $\varepsilon_{pp}$ data, rounded to one ppm, are {96, 193, 250}, while {94, 194, 243} ppm was reported some time ago for $\delta_{pp}$ in the α-helical form of poly(L-alanine) [42]. The values of α*,* β*,* γ angles (see Materials and Methods for their definition) show that the investigated ^13^C CST is approximately orthogonal in the coordinate system with one of its axes lying in the peptide plane, another axis being parallel to the C=O bond vector, and the remaining axis being perpendicular to the peptide plane.

**Figure 4:**
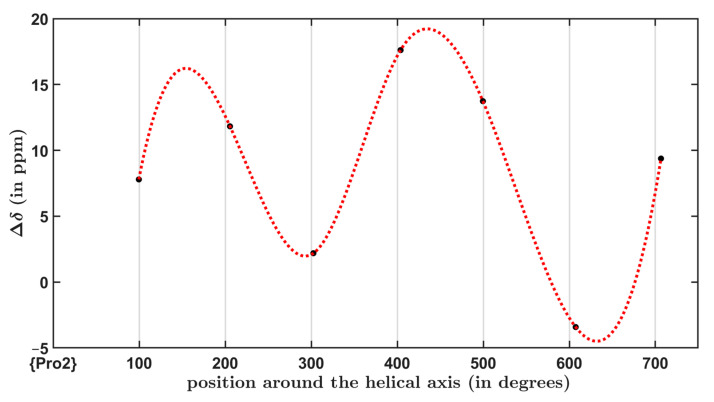
The dependence of the total ^13^C chemical shift anisotropy upon the rotation angle in the ALM-18 model. The simulated functional dependence is expressed by Equation (2), while the datapoints are described in Section 2.3.

For a helical peptide in magnetically oriented lipid bilayers, the $\delta_{∥}$. and $\delta_{⊥}$^13^C chemical shifts in carbonyl carbons can be measured ($\delta_{∥}$ and $\delta_{⊥}\;$ represent rotationally averaged values for orientations originally specified in reference [18] and are not repeated here), and the total ^13^C chemical shift anisotropy, Δδ, is then obtained as $\Delta \delta =\delta_{∥}-\delta_{⊥}$. Importantly, an Δδ expression can be developed into a sum of the axially symmetric contribution, $\delta^{\mathrm{aniso}}$, and of the orientation-dependent term connected with the ρ, τ  angles from Section 2.2. The axially symmetric contribution is $\delta^{\mathrm{aniso}}=\delta_{22}-\left(\delta_{11}+\delta_{33}\right)/2$, as it should be noted that an analysis of these Δδ measurements assumes that the direction of the $\delta_{22}\;$ component is parallel to the helix axis of an investigated peptide [18]. It should also be noted that the direction of the $\delta_{22}\;$ component is almost collinear with the C=O bond vector (see values of the β angle in Table 3). Theoretical values of the $\delta^{\mathrm{aniso}}$ for ALM-E18 model designated $\varepsilon^{\mathrm{aniso}}$ in Table 3 are $\varepsilon^{\mathrm{aniso}}=\varepsilon_{22}-\left(\varepsilon_{11}+\varepsilon_{33}\right)/2$, and their variation is much higher than for $\varepsilon^{\mathrm{iso}}$ data. Namely, the $\varepsilon^{\mathrm{aniso}}$ values lie in the interval spanning more than 36 ppm. The orientation-dependent contribution to the total chemical ^13^C chemical shift anisotropy is shown in Equation (2) in the Materials and Methods section. In short, this term describes an oscillation of the chemical shift anisotropy. This oscillation is expressed in terms of the amplitude and phase data, where amplitude and phase depend on the values of ρ and τ, respectively. The oscillatory pattern of Δδ values is of importance in structural studies of membrane-bound biomolecules [22]. Here, the results of the PW DFT calculations obtained for ALM are combined with τ = 8.0° (see Section 2.2. for background) to model the ^13^C chemical shift anisotropy oscillations in a relatively regular α-helical fragment for sites ranging from Aib3 to Val9. The site-specific ρ values are used, which were obtained by parametrizing the helix as outlined in Appendix A, together with the relevant data from Table 3. This simulation is graphically presented in Figure 4. It shows the dependence of the total ^13^C chemical shift anisotropy upon the respective azimuthal position of the carbonyl carbons with respect to the helix axis (the grid lines indicate 100° spacing, which would be present in an ideal α-helix, while the dotted line represents a cubic spline connecting datapoints). By performing this simulation for a set of the tilt angles, it might be possible to extract an accurate τ value, which would be consistent with the experimentally observed chemical shift anisotropy oscillations in an actual ALM sample.

## 3. Materials and Methods

### 3.1. The Periodic DFT Calculations 

The PW DFT calculations were performed to treat an investigated crystal as an infinite system while using the pseudopotential scheme [43,44,45], as implemented in the CASTEP 16.1 code. Input files were prepared using the Materials Studio 2019 [46]. Crystalline structures of ALM [26], glycylglycine [47], glycylglycine nitrate [48], and glycylglycine hydrochloride hydrate [49] were considered. In their CASTEP calculations, the ultrasoft on-the-fly-generated pseudopotentials [50] were applied, together with settings corresponding to the “Fine” accuracy level of the Materials Studio software. First, all internal coordinates and unit cell parameters of these crystals were optimized using the Perdew–Burke–Erzerhof (PBE) exchange-correlation DFT functional [51], in combination with the Tkatchenko–Scheffler dispersion-correction strategy [52]. Subsequently, the chemical shielding tensors were obtained for the optimized structures using the PBE functional and gauge-including projector augmented wave (GIPAW) approach [53,54]. This methodology was thoroughly tested [55]. The MAGRES files can be obtained from the corresponding author upon request.

Angles α, β, γ describe the orientation of the chemical shielding tensors, which were predicted by the PBE-GIPAW method, in the molecular frame of crystalline ALM after the PW PBE structural optimization. These angles are expressed using atom coordinates in the relevant peptide plane, together with the eigenvectors $\xi_{1},\;\xi_{2},\;\xi_{3}$ associated with eigenvalues $\sigma_{11},\;\sigma_{22},\;\sigma_{33}$, respectively, of the investigated chemical shielding tensor ($\sigma_{11}\geq \sigma_{22}\geq \;\sigma_{33})$. For the ^15^N amidic nuclei, N_amid_, the related {N_amid_, H_amid_, C_α_} coordinates are used here to define the peptide plane, $P_{N}$. Then, angle α is obtained by projecting $\xi_{3}$ on $P_{N}$, γ is taken between $\xi_{2}$ and a normal to $P_{N}$, and  β is the angle between $\xi_{3}$ and the N_amid_–H_amid_ bond vector. For the ^13^C carbonyl nuclei, C’, the peptide plane is denoted as $P_{C}$ and defined using {C’, O’, C_α_} coordinates. In this case, the angle α is obtained by projecting $\xi_{2}$ on $P_{C}$, γ is taken between $\xi_{1}$ and a normal to $P_{C}$, and  β is the angle between $\xi_{2}$ and the C’–O’ bond vector. 

### 3.2. Simulations of the SSNMR Spectral Data 

The parallel component of the ^15^N CST, $\delta_{∥}$, which was discussed in Section 2.2, is

(1) $$\delta_{∥}=\delta_{11}\sin^{2}\tau +\delta_{33}\cos^{2}\tau$$

where $\delta_{11}$ and $\delta_{33}\;$ are principal elements of the CST, and τ is the tilt angle. Details of the related model are given in reference [56]. It should be noted that the Euler angles α, β in Equation (9) of Bechinger and Sizun [56] are set here to 0° and to a value of τ, respectively.

The total ^13^C chemical shift anisotropy, Δδ, which was discussed in Section 2.3, is

(2) $$\Delta \delta =3/2\;\sin^{2}\tau \;\left(\delta_{11}\cos^{2}\rho +\delta_{33}\sin^{2}\rho -\delta_{22}\right)+\delta_{22}-\left(\delta_{11}+\delta_{33}\right)/2$$

where $\delta_{11},\;\;\delta_{22},\;\;\delta_{33}\;$ are principal elements of the ^13^C CST, and ρ and τ are the azimuthal rotation and tilt angles, respectively. Clearly, the orientation-dependent contribution to Δδ is given by the term with a 3/2 prefactor. 

## 4. Conclusions

The fully periodic PW DFT calculations can support the SSNMR investigations of even large crystalline oligopeptides, such as the ALM studied here. Specifically, these calculations reliably describe a variation in the CSTs along the peptide backbone. This variation is directly taken into account in simulations of the anisotropic ^15^N and ^13^C chemical shift components, namely, by employing the CST parameters and the structural data predicted for specific amino acid sites. Then, the effect of this variation can be assessed on the angles that specify the alignment of helical peptides in membranes. The applications of this approach to other antimicrobial peptides, and other SSNMR data (in particular, the ^17^O SSNMR parameters [57,58]), are expected.

## Figures and Tables

**Table 1:** Structural features of ALM-18 model.

**Residue**	φ **(Degrees)**	ψ **(Degrees)**	|φ+ψ| **(Degrees)**	H-Bonding Involvement
Aib1	−49	−44	−93	→Aib5
Pro2	−65	−34	−99	→Aib6
Aib3	−57	−49	−106	→Gln7
Ala4	−66	−43	−109	→Aib8
Aib5	−54	−51	−105	→Val9, Aib1←
Ala6	−68	−37	−105	→Aib10, Pro2←
Gln7	−61	−45	−106	→Gly11, Aib3←
Aib8	−56	−46	−102	Ala4←
Val9	−64	−50	−114	→Leu12, Aib5←
Aib10	−53	−43	−96	→Aib13, Ala6←
Gly11	−66	−18	−84	Gln7←
Leu12	−94	−13	−107	→Aib16, Val9←
Aib13	−51	−42	−93	→Aib17, Aib10←
Pro14	−68	−23	−91	→Glu18
Val15	−67	−48	−115	→Gln19
Aib16	−54	−50	−104	→Phl20, Leu12←
Aib17	−57	−44	−101	→Phl20 hydroxyl, Aib13←
Glu18	−61	−37	−98	Pro14←
Gln19	−78	−35	−113	Val15←
Phl20	−141	― ^1^	― ^1^	Aib16←, Aib17←

^1^ undefined.

**Table 2:** Predicted values of the ^15^N SSNMR parameters of amidic nitrogens in ALM-E18 model.

**Residue**	$\varepsilon^{\mathrm{iso}}$ **(ppm)**	$\varepsilon_{11}$ **(ppm)**	$\varepsilon_{22}$ **(ppm)**	$\varepsilon_{33}$ **(ppm)**	α **(Degrees)**	β **(Degrees)**	γ **(Degrees)**
Aib1	136.7	77.1	91.5	241.7	2.5	14.1	12.9
Pro2	133.6	47.7	125.4	227.6	― ^1^	― ^1^	― ^1^
Aib3	120.3	63.5	75.0	222.3	7.0	14.8	28.7
Ala4	117.7	48.3	81.7	223.2	1.1	16.2	17.2
Aib5	125.7	68.2	78.4	230.5	4.3	13.1	31.3
Ala6	116.6	53.7	75.5	220.7	2.9	18.2	16.1
Gln7	119.7	56.8	76.0	226.3	4.6	18.2	30.1
Aib8	127.8	68.5	78.6	236.4	4.2	12.9	51.5
Val9	114.3	51.6	78.0	213.2	1.6	17.6	12.0
Aib10	127.8	69.0	81.4	233.1	3.1	14.6	48.0
Gly11	101.2	45.0	55.7	202.9	4.0	23.8	45.2
Leu12	116.5	48.7	77.1	223.8	4.1	18.8	36.7
Aib13	131.5	71.5	86.0	237.1	2.1	14.6	70.0
Pro14	131.0	50.8	118.5	223.7	― ^1^	― ^1^	― ^1^
Val15	116.4	57.6	71.6	220.0	5.3	20.1	48.5
Aib16	125.8	62.9	83.4	231.1	1.6	10.9	17.8
Aib17	122.6	65.5	79.3	223.0	4.6	14.4	10.1
Glu18	115.8	56.7	73.1	217.5	4.9	20.8	13.9
Gln19	117.8	52.7	75.2	225.4	2.0	19.1	24.3
Phl20	112.0	56.9	65.8	213.3	5.4	19.9	15.8

^1^ undefined.

**Table 3:** Predicted values of the ^13^C SSNMR parameters of carbonyl carbons in ALM-E18 model.

**Residue**	$\varepsilon^{iso}$ **(ppm)**	$\varepsilon_{11}$ **(ppm)**	$\varepsilon_{22}$ **(ppm)**	$\varepsilon_{33}$ **(ppm)**	α **(Degrees)**	β **(Degrees)**	γ **(Degrees)**	$\varepsilon^{aniso}$ **(ppm)**
Aib1	178.0	102	181.9	250.0	1.2	4.8	1.4	5.9
Pro2	180.5	97.2	189.0	255.2	1.7	4.0	4.2	12.8
Aib3	180.2	100.3	187.0	253.4	1.2	2.6	1.4	10.2
Ala4	179.4	98.5	188.8	250.9	0.5	2.3	1.5	14.1
Aib5	180.0	101.0	183.0	256.0	1.2	1.8	2.2	4.5
Ala6	179.7	95.9	193.2	249.9	3.1	3.1	3.4	20.3
Gln7	178.1	97.0	189.0	248.2	0.3	2.0	1.7	16.4
Aib8	179.5	101.4	178.4	258.6	0.2	1.9	1.9	−1.6
Val9	179.1	96.8	187.0	253.5	0.6	0.8	0.9	11.9
Aib10	182.1	99.6	189.3	257.3	1.4	1.4	2.6	10.9
Gly11	171.7	94.4	166.8	253.8	2.2	3.0	2.3	−7.3
Leu12	180.9	97.3	198.7	246.8	0.8	4.2	0.8	26.6
Aib13	178.2	102.1	182.7	249.8	0.5	5.3	1.5	6.8
Pro14	179.1	94.4	190.0	252.8	0.7	1.3	3.0	16.4
Val15	177.9	97.9	182.9	253.0	0.3	2.2	0.7	7.4
Aib16	178.9	100.5	182.5	253.7	0.1	2.1	2.1	5.3
Aib17	179.2	98.7	188.6	250.5	1.5	3.8	2.9	14.0
Glu18	177.6	92.8	196.9	243.1	3.3	5.6	4.0	28.9
Gln19	179.2	95.6	186.8	255.2	1.8	3.8	3.1	11.4

**Figure A1:**
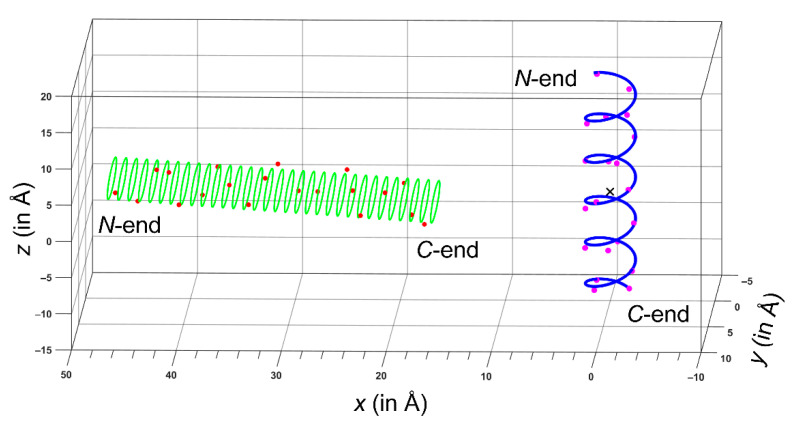
The cylinder fitted to original C_α_ positions, and the parametrized helix, together with projected positions of carbonyl carbons of the ALM backbone.

## Data Availability

The data presented in this study are available in the article and in the Supplementary Materials.

## Supplementary Materials

The following are available online at https://www.mdpi.com/article/10.3390/antibiotics10101265/s1, Table S1: Raw data used to describe the relationship between the ^13^C chemical shifts and shieldings of carbonyl carbons in three small compounds, Figure S1: Plot of the relationship between the ^13^C chemical shifts and shieldings data of carbonyl carbons from Table S1, ‘alm.cif’ and ‘alm.pdb’ files containing the structure described in Section 2.1.

## Appendix A

There are a number of approaches for fitting a helical curve to molecular structures (see the recent survey in reference [59]). Nevertheless, a simple method was implemented to obtain the full parametrization of an irregular helix of ALM. The procedure begins by fitting the coordinates of C_α_ atoms to a right circular cylinder. This is performed in the original coordinate system of ALM. The fitted parameters of the cylinder are: its center, C; its axis that is a unit vector, $\vec{a}$; and its radius, R. Using the “fmincon” nonlinear constrained optimization method in Matlab^®^, the following values are obtained: C at [29.37, 6.37, 4.46]; $\vec{a}\;$= (0.996, –0.018, 0.085); and  R = 2.37 Å. The resulting fit is represented in Figure A1 by thin green circles, while the C_α_ datapoints are shown as small red balls. The accuracy of this approximation can be described by an average distance, d, between the actual C_α_ positions and the nearest points on the fitted cylinder. Expectedly, this accuracy is not high (d = 0.32 Å), because the ALM helix is irregular. In the next step, the ALM coordinates are translated so that their centroid is at the point O with coordinates [0, 0, 0], and then are rotated so that their new z axis direction coincides with the direction of $\vec{a}$. The rotation matrix (not shown) was found using our INFOR code [60]. In this new coordinate system, the positions of carbonyl carbons are projected on the fitted cylinder. In Figure A1, these positions are shown as magenta balls (the subset of these projected positions was used to obtain the ρ angles discussed in Section 2), and the point O is marked with black cross. In the final step, an approximate parametric representation, h(t), of the helix wrapped around the fitted cylinder, is found. This is given by Equation (A1) and shown as the thick blue line in Figure A1, where the parameter t  goes from 0 to 10.5π.

(A1) $$h\left(t\right)=\left(\begin{array}{c} 0\\ 0\\ -14.95 \end{array}\right)+\left(\begin{array}{ccc} -0.6536 & 0.7568 & 0\\ -0.7568 & -0.6536 & 0\\ 0 & 0 & 1 \end{array}\right)\left(\begin{array}{c} R\cos\left(t\right)\\ R\sin\left(t\right)\\ 0.9072\;t \end{array}\right)$$
