# Supplementary material for: Modeling the Structure of Crystalline Alamethicin and Its NMR Chemical Shift Tensors
Source: Antibiotics (Basel). 2021 Oct 18;10(10):1265. doi: 10.3390/antibiotics10101265 (PMC8532780; doi:10.3390/antibiotics10101265)

Supporting Materials to "Modeling the Structure of Crystalline Alamethicin and Its NMR Chemical Shift Tensors" by Czernek & Brus (*Antibiotics* **2021**)

Table S1. Raw data used to describe the relationship between the ^{13}C chemical shifts and shieldings of carbonyl carbons in three small compounds.

compound	tensor component	δ_{ii} (in ppm)	σ_{ii} (in ppm)
glycylglycine (G2)	least shielded	242.3	–68.3154
	mid shielded	173.8	–2.8681
	most shielded	88.2	85.0480
G2 * HNO_3	least shielded	248.1	–73.2241
	mid shielded	167.8	3.4246
	most shielded	89.1	84.6969
G2 * HCl * H_2O	least shielded	242.1	–73.0879
	mid shielded	177.1	–5.1053
	most shielded	87.9	85.7582

Figure S1. Plot of the relationship between the ^{13}C chemical shifts and shieldings data of carbonyl carbons from Table S1.

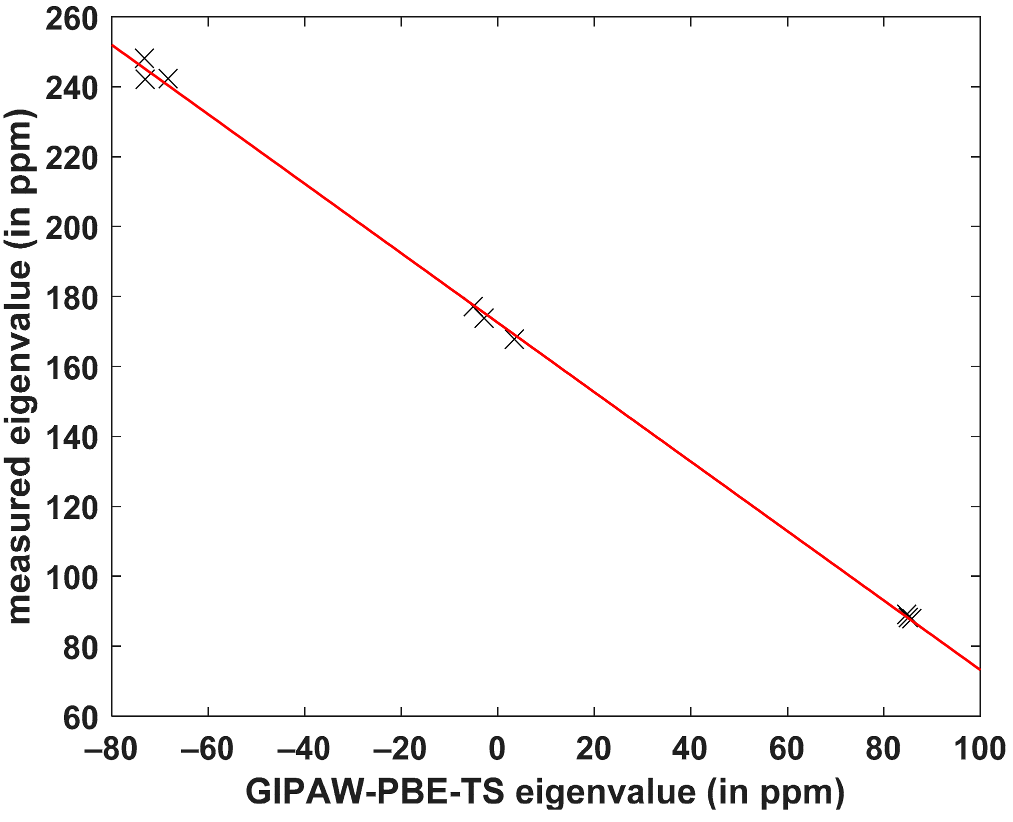

Supplement: Supplementary file 1 [file antibiotics-10-01265-s001.zip › SI.pdf]
